# A conceptual model for students’ satisfaction with team-based learning using partial least squares structural equation modelling in a faculty of life sciences, in the United Kingdom

**DOI:** 10.3352/jeehp.2019.16.36

**Published:** 2019-11-13

**Authors:** Andrea Manfrin, Bugewa Apampa, Prabha Parthasarathy

**Affiliations:** 1 School of Pharmacy and Biomedical Science, Faculty of Clinical & Biomedical Sciences, University of Central Lancashire, Preston, UK; 2 Sussex Pharmacy, School of Life Sciences, University of Sussex, Falmer, UK; 3 Faculty of Medicine, Imperial College, London, UK; Hallym University, Korea

**Keywords:** Least-squares analysis, Personal satisfaction, Problem-based learning, Students, United Kingdom

## Abstract

**Purpose:**

Students’ satisfaction is an essential element in higher education. This study aimed to identify paths and predictive power of students’ satisfaction during team-based learning (TBL) activities in the faculty of life sciences using partial least squares structural equation modelling (PLS-SEM).

**Methods:**

In 2018–2019, at the University of Sussex (Falmer, UK), 180 life science students exposed to TBL were invited to participate in the study. Team-Based-Learning-Student-Assessment-Instrument was used. A conceptual model was developed for testing six hypotheses. H1: What was the effect of TBL on student satisfaction? H2: What was the effect of lectures on student satisfaction? H3: What was the effect of TBL on accountability? H4: What was the effect of lectures on accountability? H5: What was the effect of accountability on student satisfaction? H6: What were the in-sample and out-of-sample predictive power of the model? The analysis was conducted using the PLS-SEM approach.

**Results:**

Ninety-nine students participated in the study giving a 55% response rate. Confirmatory tetrad analysis suggested a reflective model. Construct reliability, validity, average extracted variance, and discriminant validity were confirmed. All path coefficients were positive, and 5 were statistically significant (H1: β=0.587, P<0:001; H2: β=0.262, P<0.001; H3: β=0.532, P<0.001; H4: β=0.063, P=0.546; H5: β=0.200, P=0.002). The in-sample predictive power was weak for Accountability, (R^2^=0.303; 95% confidence interval [CI], 0.117–0.428; P<0.001) and substantial for student satisfaction (R^2^=0.678; 95% CI, 0.498–0.777; P<0.001). The out-of-sample predictive power was moderate.

**Conclusion:**

The results have demonstrated the possibility of developing and testing a TBL conceptual model using PLS-SEM for the evaluation of path coefficients and predictive power relative to students’ satisfaction.

## Introduction

Team-based learning (TBL) is an evidence-based collaborative learning and teaching strategy designed around units of instruction, known as “modules,” that are taught in a three-step cycle: preparation, in-class readiness assurance testing, and application-focused exercise. A class typically includes one module.; the primary learning objective of TBL is to go beyond simply covering content and focus on ensuring that students have the opportunity to practise using course concepts to solve problems. Structural equation modelling (SEM) represents a group of statistical techniques that have become very popular in business and social sciences search [1]. Partial least squares structural equation modelling (PLS-SEM) is a prediction-oriented variance-based approach that focuses on endogenous target constructs in the model and aims at maximising their explained variance (e.g., looking at the coefficient of determination (R^2^) value) [2]. PLS-SEM has been used to explore pharmacists’ job satisfaction and the effects of different indicators on job satisfaction [3], and more recently to explore the influence of pharmacists’ expertise on the prescribing decisions of physicians [4]. A few studies conducted in the United Kingdom analysed the use of TBL with the team-based learning students assessment instruments (TBL-SAI) [5,6]. To the best of our knowledge PLS-SEM has not been used to evaluate students’ accountability, preference for TBL or lectures and satisfaction as measured using the TBL-SAI in the United Kingdom.

It aimed to identify paths and predictive power of students’ satisfaction during team-based-learning activities in the faculty of life sciences using PLS-SEM.

## Methods

### Ethics statement

The study was conducted in accordance with the Helsinki Declaration of 1975 as revised in 2008, and received ethical approval from the Life-Sciences-Psychology-Cluster-based-Research Ethics Committee of the University of Sussex on 9/11/2018 (ref: ER/PP225/1) for pharmacy and biomedical students, and on 15/02/2019 (ref: ER/AAM2078/2) for foundation year science students. Informed consent was obtained from all individual participants included in the study. During the final TBL teaching session, students were invited to complete an online questionnaire delivered through a web platform called Qualtrics available from https://www.qualtrics.com. All data were treated following the requirements of the Data Protection Act (2018) and/or General Data Protection Regulation (2016).

### Study design

This is a cohort study used to test a methodological approach.

### Population

Three groups of students at the University of Sussex (Falmer, UK) were involved in this research: year 1 pharmacy students, year 2 biomedical students, foundation year science students enrolled in an Introduction to Clinical Sciences module. During the academic year 2018–2019, pharmacy and biomedical students were exposed to TBL activities during term 1, while foundation students were exposed in term 2 because their module was delivered in term 2.

### Research instrument

The TBL-SAI instrument is a well-recognised instrument used for assessing students’ accountability preferences for TBL or lectures and satisfaction. The instrument was developed by Mennenga [7] in 2012, who approved its use. Initially, all the TBL-SAI questions (n=33) were included in the analysis; however, questions with lower loading coefficients were removed after each iteration (n=13); therefore, it was decided to include the questions with outer loading coefficients closer or above 0.7. Twenty questions were included in the final model and analysed (Supplement 1).

### Conceptual model

A path model is a diagram that displays the hypotheses and variable relationships to be estimated in an SEM analysis. The proposed model was analysed according to the flow chart developed by Sarstedt and Ringle [8] in 2017. The analysis of the model was conducted in different stages: (1) the assessment of the type of model: reflective or formative; (2) the use of the measurement model (outer model) which reveals the relationships between latent indicators and their variables; (3) the use of the structural model (inner model) which comprises the evaluation of the relationships between the latent variables; and (4) the use of PLS predict to evaluate the predictive power of the model.

**Fig. 1.**
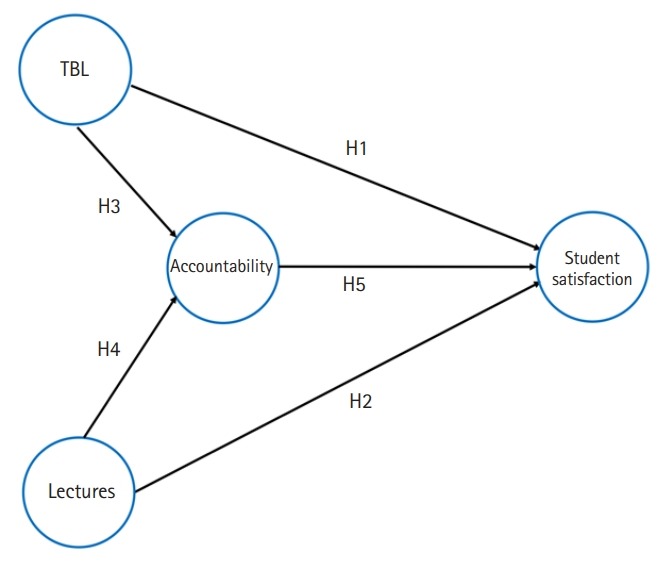
Conceptual model. The arrows are connecting the circles, and the direction of the arrows represent the hypothesis that we were going to test. TBL, team-based learning.

The conceptual model summarises the research questions (hypothesis) that this study was aiming to test (Fig. 1): (1) hypothesis 1 (H1): What was the effect of TBL on student satisfaction?; (2) hypothesis 2 (H2): What was the effect of lectures on student satisfaction?; (3) hypothesis 3 (H3): What was the effect of TBL on accountability?; (4) hypothesis 4 (H4): What was the effect of lectures on accountability?; (5) hypothesis 5 (H5): What was the effect of accountability on student satisfaction?; (6) hypothesis 6 (H6): What were the in-sample and out-of-sample predictive power of the model?

### Study power

A post hoc power calculation was conducted using G*Power ver. 3.1.9.3 (Heinrich-Heine-Universität Düsseldorf, Düsseldorf, Germany; http://www.gpower.hhu.de/) [9]. A 2 tails t-test was conducted using a linear multiple regression, with a fixed model and a single regression coefficient applying the following information: the number of students who took part in the study (n=99), the number of predictors (n=7), the effect size (f^2^=0.15), and the probability of alpha error (0.05). The power of the study obtained was of 97%, with a degree of freedom of 91, a critical t=±1.98, and a non-centrality parameter *δ*=3.85.

### Data collection cleaning and analysis

Data were collected using an online platform, then imported into IBM SPSS ver. 25.0 (IBM Corp., Armonk, NY, USA) for data cleaning. The Kolmogorov-Smirnov test showed that the data were not normally distributed. Sarstedt et al. [10] in 2016 suggested that PLS-SEM shows higher robustness when handling non-normally distributed data. Therefore, the SPSS data set was exported as a CSV file and then uploaded onto SmartPLS ver. 3.2.8 (SmartPLS GmbH; https://www.smartpls.com), which is a variance-based structural equation model suitable for non-normally distributed data (Dataset 1).

### Procedure for model assessment and statistical analysis

The use of PLS-SEM allowed the analysis of the linear relationships between the latent constructs and the latent variables. Furthermore, PLS-SEM enabled the testing of several relationships instead of analysing each relationship individually. P-values <0.05 or 0.1 were considered statistically significant according to the different procedures. The model assessment and data analysis are fully explained in the Supplement 2.

## Results

### Demographics

The number of students invited was 180; 26 Pharmacy (year 1), 90 Biomedical Science (year 2), 64 Introduction to Clinical Sciences (foundation year). Ninety-nine students participated in the study giving an overall response rate of 55%. Over 70% of the student population was female, the higher percentage (92.90) was in the 16–24-year range, A-level and IB were the most common entry qualifications, others (e.g., Romanian Baccalaureate, BTEC [Business and Technology Education Council] diploma), and 96% were from the United Kingdom/European Union (Table 1).

### Confirmatory tetrad analysis

The results of the confirmatory tetrad analysis (CTA) showed that for each construct all the values in the low adjusted confidence interval (CI) were negative, while in the up adjusted CI were positive, meaning that zero lays between these values, suggesting that the model was reflective (Table 2).

### Reflective measures

**Fig. 2.**
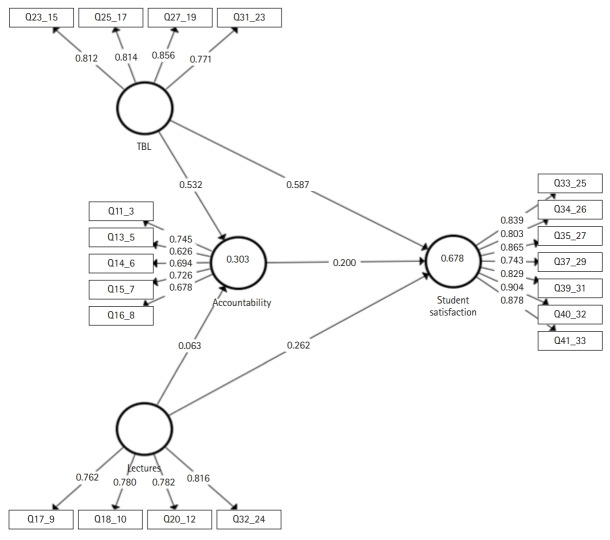
Path model (reflective). The values inside the circles represent the coefficient of determination (R^2^). The values overlapping the arrows pointing towards the rectangles represent the outer loading coefficients. The values overlapping the arrows between the circles (constructs) represent the path coefficients (standardised beta=beta coefficients).

Fig. 2 shows the path model generated using the PLS algorithm. The circles represent the constructs (latent variables) the squares represent the indicators, and the arrows pointed towards the indicators show the reflective type of measures.

### Evaluation of the measurement model (outer model)

#### Reliability and validity

All the values presented in Table 3 show that that model has construct reliability and validity. Only three out of 11 loading coefficients were just below 0.70 (Q11, Q20, Q24). Cronbach’s α, ⍴_A_, and ⍴_C_, were all above the threshold while the average variance extracted (AVE) for accountability was below the threshold but was considered acceptable. The lower values identified in the loadings and AVE were accepted due to the exploratory nature of the study.

#### Discriminant validity

Five out of 6 heterotrait-monotrait ratio (HTMT) values were <0.85, using the more conservative approach HTMT_85_, but all of them were <1 using HTMT_90_; furthermore, the HTMT values shown in the upper bond of the 95% CI and 95% CI BCa (bias-corrected and accelerated bootstrap) were also <1, meaning that discriminant validity was established (Table 4). The results presented in Tables 3 and 4 confirmed that the measurements of the reflective model were valid and reliable.

### Evaluation of the structural model (inner model)

#### Co-linearity among constructs

The variance inflation factor (VIF) values were: accountability-student satisfaction, 1.435; lectures-accountability, 1.065; lectures-student satisfaction, 1.071; TBL-accountability, 1.064; and TBL-student satisfaction, 1.417. The analysis of the co-linearity among constructs showed that all the VIF values were well below 3; therefore, the inner model did not present co-linearity issues.

### Testing the hypotheses (H1 to H6)

#### In-sample prediction: significance and relevance of path coefficients

Path coefficients also called standardised beta (ß) usually vary between -1 and +1. The higher the absolute value, the stronger is the predictive relationship between the constructs. The hypothesis that we tested (H1 to H5) showed that all path coefficients had a positive sign meaning that they had a positive influence on the construct (e.g., if the TBL increased, student satisfaction increased). The higher value was represented by TBL-student satisfaction (ß=0.587, t=8.398, P<0.001), the second higher value was TBL-accountability (ß=0.532, t=6.667, P<0.001); the lower value was lectures-accountability (ß=0.063, t=0.604, P=0.546) which was also the only one non-statistically significant measure (Table 5).

The significance and relevance of the path coefficients were also evaluated, looking at the effects (Table 6). The higher effect was represented by the total effect of TBL+accountability+student satisfaction (0.693), while the lower effect by the direct effect of lectures-accountability (0.063).

#### In-sample predictive power

R^2^ is a measure of the model explanatory power and represents the amount of variance in the endogenous construct (e.g., student satisfaction) explained by all the exogenous constructs linked to it (e.g., TBL, lectures). R^2^ ranges between 0 and 1 with a larger value indicating higher levels of explanatory power. The coefficients of determination (R^2^) were calculated for obtaining an in-sample prediction. The R^2^ for accountability was 0.303 showing a weak predictive power, while the R^2^ (0.678) of student satisfaction was closer to the substantial predictive power (Table 7). The effect size (f^2^) shows how strong one exogenous construct contributes to explaining a certain endogenous construct in terms of R^2^. A weak effect is 0.02≤f^2^<0.15, moderate effect 0.15≤f^2^<0.35, and strong effect f^2^≥0.35. The value of f^2^ for accountability-student satisfaction was 0.086 (95% CI, 0.009–0.250; P=0.209), lectures-accountability 0.005 (95% CI, 0.000–0.094; P=0.848), lectures-student satisfaction 0.2 (95% CI, 0.063–0.477; P=0.069), TBL-accountability 0.381 (95% CI, 0.158–0.791; P=0.023) and for TBL-student satisfaction 0.728 (95% CI, 0.308–1.333; P=0.008). Therefore, student satisfaction has a moderate/substantial predictive power, while accountability has weak predictive power (Table 7).

#### Out-of-sample predictive power

The predictive relevance (Q^2^) in the PLS model was confirmed by the Q^2^ values which were all >0, therefore meaningful, while in one case in the linear model (LM) model Q^2^ was <0. The interpretation of the output of PLSpredict was conducted by a comparative analysis looking at whether the PLS analysis compared to the LM analysis yields higher prediction errors in terms of root mean squared error (RMSE). Hair et al. [11] in 2019 suggested that higher RMSE values in the PLS output for all meant no predictive power; for the majority, low predictive power; for the minority or the same number, medium predictive power; and for none of the indicators, high predictive power. Table 8 showed that all RMSE (PLS) values, except for one (Q39_31), were lower than the RMSE (LM). Therefore, this model has a moderate/high out-of-sample predictive power.

## Discussion

### Key results

This study aimed to identify paths and predictive power of students’ satisfaction during TBL activities in the faculty of life sciences. The student population was a mix of 3 different disciplines: pharmacy, foundation year and biomedical sciences. The highest percentage of students (53%) was in biomedical science and the lowest in pharmacy (18%). The researchers developed a conceptual model for visualising the connections among the inner variables (latent variables) which displayed the hypotheses and the variables relationships estimated by the PLS-SEM analysis. The analytical approach adopted was the one suggested by Sarstedt and Ringle [8] in 2017. Six hypotheses were formulated and tested. The CTA showed the reflective structure of the model. The model was reliable, consistent and had discriminant validity suggesting that the results confirmed that the hypothesised structural paths were real, and not a mere result of statistical discrepancies. The AVE of accountability was just below the 0.5 threshold (0.483), due to the values of 3 loading coefficients, which were just <0.7. The general rule is that AVE should be ≥0.5; but if the AVE is less than 0.5 and the composite reliability is higher than 0.6, as in our case (⍴_C_=0.823), the convergent validity of the construct is still valid. The hypotheses H1 to H5 were tested using the significance and relevance of the path coefficients; all of them suggested a positive linear relationship between the variables in each hypothesis. The higher value was for TBL-student satisfaction, the lower for lectures-accountability, which was the only one non-statistically significant, suggesting that lectures did not have a statistically significant impact on accountability while TBL did. The in-sample predictive power of the model indicated that student satisfaction had a substantial predictive power showing the higher coefficient of determination (R^2^=0.678); the out-of-sample predictive power of the model was moderate.

### Interpretation

These are two very important messages because they reinforce the idea that TBL has the potential of improving student satisfaction and perhaps engagement. Cheong and Ong [12] in 2016 identified a statistically significant relationship between engagement and satisfaction, but these results were not confirmed by Pelletier et al. [13] in 2017. Urbonas et al. [3] in 2015 used PLS-SEM in their study and Q^2^ for assessing the predicting validity of the model; this study was published in 2015; therefore, the possibility of using a more enhanced analysis such as the one suggested by Shmueli et al. [14] in 2016 and then introduced into SmartPLS, such as PLSpredict, was not available [7]. Rathner and Byrne [15] in 2014 assessed the impact of TBL on student performance within the Health Science degree at La Trobe University (Australia) using SEM. Their model showed that weaker students working in strong teams could overcome their educational disadvantages. One of the limitations of this study was that the predictability of student performance was calculated only for the in-sample model.

### Strengths and limitations

This study appears, to the best of our knowledge, the first attempt of using PLS-SEM to evaluate a TBL conceptual model based on the TBL-SAI and is one of the few evaluating 3 different student populations; pharmacy, biomedical sciences and foundation degree. The model was robust, showing reliability, positive paths, and predictive power. The major limitation of this study is the small sample size (n=99) which we believe had an impact on the loading coefficients of different variables, and for this reason, we did not use all the questions on the TBL-SAI.

### Conclusion

This study has demonstrated the possibility of developing and testing a conceptual model using TBL, and the application of PLS-SEM for the evaluation of its path coefficients and predictive power as well. Nevertheless, the positive results of this study need to be taken with caution because we were not able to evaluate the model using all the questions on the TBL-SAI. Further research is needed, using a larger sample for testing and validating the model and including all the TBL-SAI questions.

## Figures and Tables

**Table 1.** Demographic profile of the respondents

Characteristic	No. (%)
Gender	
Female	71 (71.70)
Male	28 (28.30)
Age range (yr)	
16–24	92 (92.90)
25–24	4 (4.00)
35–45	1 (1.00)
>45	2 (2.00)
Entry qualification^a)^	
A level/IB	64 (64.64)
Foundation year	29 (29.29)
Others	14 (14.14)
Returning after break	4 (4.00)
Ethnicity	
White	64 (64.65)
Asian/Asian British	12 (12.12)
Black/African/Caribbean/Black British	10 (10.10)
Mixed/multiple ethnic groups	9 (9.09)
Others	3 (3.03)
Prefer not to say	1 (1.01)
Residence status	
United Kingdom/EU	95 (96.00)
Non-United Kingdom/non-EU	4 (4.00)
Discipline	
Biomedical science	52 (52.50)
Foundation: introduction to clinical sciences	29 (29.30)
Pharmacy	18 (18.20)

EU, European Union.

a) Entry qualification do not add up to 100%.

**Table 2.** Confirmatory tetrad analysis partial least squares results

Vanishing tetrads	Original sample	Bootstrap t-value	P-value	Adjusted confidence level
Accountability				
Q11_3, Q13_5, Q14_6, Q15_7	-0.005	0.372	0.710	-0.037 to 0.027
Q11_3, Q13_5, Q15_7, Q14_6	-0.030	1.696	0.090	-0.073 to 0.010
Q11_3, Q13_5, Q14_6, Q16_8	0.016	0.660	0.509	-0.040 to 0.073
Q11_3, Q14_6, Q16_8, Q13_5	-0.003	0.162	0.871	-0.050 to 0.043
Q11_3, Q14_6, Q15_7, Q16_8	0.008	0.286	0.775	-0.059 to 0.075
Lectures				
Q17_9, Q18_10, Q20_12, Q32_24	0.184	1.618	0.106	-0.034 to 0.413
Q17_9, Q18_10, Q32_24, Q20_12	0.140	1.200	0.230	-0.086 to 0.373
Student satisfaction				
Q33_25, Q34_26, Q35_27, Q37_29	0.009	0.301	0.763	-0.072 to 0.091
Q33_25, Q34_26, Q37_29, Q35_27	-0.137	1.788	0.074	-0.348 to 0.064
Q33_25, Q34_26, Q35_27, Q39_31	0.108	1.445	0.149	-0.091 to 0.310
Q33_25, Q35_27, Q39_31, Q34_26	0.066	1.875	0.061	-0.026 to 0.164
Q33_25, Q34_26, Q35_27, Q41_33	0.122	1.852	0.064	-0.052 to 0.303
Q33_25, Q34_26, Q37_29, Q39_31	-0.140	1.655	0.098	-0.375 to 0.081
Q33_25, Q34_26, Q37_29, Q41_33	0.024	0.421	0.674	-0.129 to 0.179
Q33_25, Q34_26, Q39_31, Q41_33	0.122	2.146	0.032	-0.028 to 0.279
Q33_25, Q40_32, Q41_33, Q34_26	-0.055	0.859	0.391	-0.229 to 0.114
Q33_25, Q35_27, Q37_29, Q40_32	-0.142	1.567	0.117	-0.392 to 0.097
Q33_25, Q35_27, Q41_33, Q37_29	0.057	1.643	0.100	-0.034 to 0.152
Q33_25, Q35_27, Q39_31, Q40_32	0.012	0.370	0.711	-0.072 to 0.095
Q33_25, Q37_29, Q40_32, Q39_31	0.121	1.410	0.159	-0.105 to 0.357
Q33_25, Q37_29, Q40_32, Q41_33	0.048	1.017	0.309	-0.078 to 0.176
Team-based learning				
Q23_15, Q25_17, Q27_19, Q31_23	-0.005	0.186	0.853	-0.054 to 0.045
Q23_15, Q25_17, Q31_23, Q27_19	-0.038	0.893	0.372	-0.123 to 0.043

Vanishing tetrads: tetrads equal to zero; t-value (statistics) thresholds: ±1.98; and statistically significant at P-value <0.05.

**Table 3.** Reliability and validity

Construct	Item	Loading (≥0.70)	CA (≥0.70)	⍴_A_ (≥70)	⍴_C_ (≥0.70)	AVE (≥0.50)
Accountability	Q11_3	0.745	0.734	0.745	0.823	0.483
	Q13_5	0.626				
	Q14_6	0.694				
	Q15_7	0.726				
	Q16_8	0.678				
Lecture	Q17_9	0.762	0.814	0.849	0.865	0.616
	Q18_10	0.780				
	Q20_12	0.782				
	Q32_24	0.816				
Team-based learning	Q23_15	0.812	0.929	0.934	0.943	0.703
	Q25_17	0.814				
	Q27_19	0.856				
	Q31_23	0.771				
Student satisfaction	Q33_25	0.839	0.829	0.836	0.887	0.662
	Q34_26	0.803				
	Q35_27	0.865				
	Q37_29	0.743				
	Q39_31	0.829				
	Q40_32	0.904				
	Q41_33	0.878				

Loading, outer loading coefficients; CA, Cronbach’s α; ⍴A, construct reliability measure (true reliability); ⍴_C_ (CR), composite reliability; AVE, average variance extracted.

**Table 4.** Discriminant validity

Constructs	HTMT	95% CI	95% CI BCa
Lectures: accountability	0.226	0.189–0.490	0.156–0.250
Lectures: student satisfaction	0.454	0.264–0.665	0.264–0.673
Accountability: student satisfaction	0.672	0.519–0.806	0.509–0.794
TBL: accountability	0.696	0.507–0.865	0.471–0.833
TBL: lectures	0.243	0.158–0.495	0.130–0.457
TBL: student satisfaction	0.853	0.720–0.935	0.715–0.931

HTMT, heterotrait-monotrait ratio; CI, confidence interval; BCa, bias-corrected and accelerated bootstrap; TBL, team-based learning.

**Table 5.** Path coefficients

Hypothesized path	Path (ß) coefficient	Bootstrap t-value	95% CI	95% BCa CI
TBL: student satisfaction (H1)	0.587	8.398	0.432 to 0.703	0.433 to 0.703
Lectures: student satisfaction (H2)	0.262	4.114	0.151 to 0.385	0.136 to 0.375
TBL: accountability (H3)	0.532	6.667	0.367 to 0.676	0.321 to 0.653
Lectures: accountability (H4)	0.063	0.604	-0.148 to 0.257	-0.148 to 0.254
Accountability: student satisfaction (H5)	0.200	3.042	0.065 to 0.335	0.054 to 0.316

Statistically significant at P-value <0.05; t-value (statistics) thresholds: ±1.98. CI, confidence interval; BCa, bias-corrected and accelerated bootstrap; TBL, team-based learning.

**Table 6.** Evaluation of the effects

Constructs	Direct effect	Indirect effect	Total effect	95% CI	95% CI Bca	P-value
Accountability: student satisfaction	0.200	NA	0.200	0.065 to 0.335	0.054 to 0.316	0.002
Lectures: accountability	0.063	NA	0.063	-0.1480 to 0.257	-0.1480 to 0.254	0.546
Lectures: student satisfaction	0.262	0.013	0.275	0.169 to 0.394	0.161 to 0.393	<0.001
TBL: accountability	0.532	NA	0.532	0.367 to 0.676	0.321 to 0.653	<0.001
TBL: student satisfaction	0.587	0.106	0.693	0.572 to 0.756	0.565 to 0.781	<0.001

Statistically significant at P-value <0.05 for the total effects. Direct effect, a relationship linking 2 constructs with a single arrow; Indirect effect, a sequence of relationships with at least one intervening construct involved; Total effect, the sum of the direct effect and all indirect linking 2 constructs; CI, confidence interval; Bca, bias-corrected and accelerated bootstrap; NA, not applicable; TBL, team-based learning.

**Table 7.** Coefficients of determination (R^2^)

Constructs	R^2^	Bootstrap t-value	95% CI	95% CI Bca	P-value
Accountability	0.303	3.660	0.181–0.485	0.117–0.428	<0.001
Student satisfaction	0.678	10.008	0.542–0.794	0.498–0.777	<0.001

Weak predictive power: R^2^≈0.25; moderate predictive power: R^2^≈0.50; substantial predictive power: R^2^≈0.75; t-value (statistics) threshold: ±1.96. CI, confidence interval; Bca, bias-corrected and accelerated bootstrap.

**Table 8.** Out-of-sample predictive power

Construct	Code	RMSE (PLS)	RMSE (LM)	Q² (PLS)	Q² (LM)
Accountability	Q11_3	0.720	0.719	0.167	0.168
	Q13_5	0.760	0.803	0.093	-0.014
	Q14_6	0.889	0.926	0.097	0.020
	Q15_7	0.625	0.660	0.132	0.033
	Q16_8	0.800	0.842	0.121	0.026
Student satisfaction	Q33_25	0.851	0.906	0.351	0.265
	Q34_26	0.838	0.895	0.414	0.332
	Q35_27	0.684	0.713	0.496	0.451
	Q37_29	0.804	0.861	0.275	0.167
	Q39_31	0.580	0.541	0.616	0.667
	Q40_32	0.725	0.754	0.445	0.400
	Q41_33	0.692	0.738	0.419	0.339

RMSE, root mean squared error; PLS, partial least squares; LM, linear model; Q2, predictive relevance.

## Footnotes

**Data availability** Data files are available from https://doi.org/10.7910/DVN/P2DNY1 Dataset 1. Data file & data dictionary.

## Supplementary materials

Data files are available from https://doi.org/10.7910/DVN/P2DNY1

Supplement 1. Constructs, code and statements included in the model.

Supplement 2. Procedure for model assessment and data analysis.

Supplement 3. Audio recording of the abstract.
