# Supplementary material for: A conceptual model for students’ satisfaction with team-based learning using partial least squares structural equation modelling in a faculty of life sciences, in the United Kingdom
Source: J Educ Eval Health Prof. 2019 Nov 13;16:36. doi: 10.3352/jeehp.2019.16.36 (PMC6887653; doi:10.3352/jeehp.2019.16.36)
Supplement: Supplementary file 2 — Supplement 1. Constructs, code and statements included in the model. [file jeehp-16-36-suppl1.docx]

**Supplement 1. Constructs, code and statements included in the model**

| **Construct** | **Code** | **Statement** |
| --- | --- | --- |
| Accountability | Q11_3 | I contribute to my team members’ learning |
|  | Q13_5 | My team members expect me to assist them in their learning |
|  | Q14_6 | I am accountable for my team’s learning |
|  | Q15_7 | I am proud of my ability to assist my team in their learning |
|  | Q16_8 | I need to contribute to my team’s learning |
| Lectures | Q17_9 | During a traditional lecture, I often find myself thinking of non- related things |
|  | Q18_10 | I am easily distracted during a traditional lecture |
|  | Q20_12 | I am more likely to fall asleep during lectures than during TBL activities |
|  | Q32_24 | After listening to a lecture, I find it difficult to remember what the instructor talked about in class |
| TBL | Q23_15 | I easily remember what I have learnt while working in a team |
|  | Q25_17 | TBL helps me recall past information |
|  | Q27_19 | I remember information for longer when I go over it with Team members in the tRAT (team readiness assurance test) |
|  | Q31_23 | I do better on exams when we use TBL to cover the material |
| Student Satisfaction | Q33_25 | I enjoy Team-based Learning activities |
|  | Q34_26 | I learn better in a team setting |
|  | Q35_27 | I think Team-based Learning activities is an effective approach to learning |
|  | Q37_29 | Team-based learning activities are fun |
|  | Q39_31 | I think Team-based learning activities help improve my grade |
|  | Q40_32 | I have a positive attitude towards Team-based learning activities |
|  | Q41_33 | I have had a good experience with Team-based learning activities |
