# Supplementary material for: A conceptual model for students’ satisfaction with team-based learning using partial least squares structural equation modelling in a faculty of life sciences, in the United Kingdom
Source: J Educ Eval Health Prof. 2019 Nov 13;16:36. doi: 10.3352/jeehp.2019.16.36 (PMC6887653; doi:10.3352/jeehp.2019.16.36)
Supplement: Supplementary file 3 — Supplement 2. Procedure for model assessment and data analysis. [file jeehp-16-36-suppl2.docx]

**Supplement 2. Procedure for model assessment and data analysis**

**Confirmatory tetrad analysis**

The initial approach was to determine whether the model was formative or reflective. Confirmatory tetrad analysis (CTA) is a method of testing and comparing the fit of an SEM-based on tetrads (difference in the product of pairs of covariance observed variables). CTA PLS was used selecting a two tails test, parallel processing, 5,000 iterations bootstrap and alpha 0.1. Then the evaluation of the model was conducted using the reflective model (steps 1 and 2) and the structural model (steps 3 to 6).

**The outer model (reflective)**

Step 1: An iterative algorithm with 300 iterations (PLS algorithm) was used to determine the internal consistency and reliability using Cronbach's alpha ≥0.70 as the lower bound, composite reliability (CR≥0.70) using Dijkstra-Henseler’s rho_A_ (⍴_A_) as the indicator of true reliability, and Dillon-Goldstein’s rho_C_ (⍴_C_) as the upper bound. Convergent validity was assessed using average variance extracted (AVE≥0.50), the outer loading coefficients with a value closer or 0.7 were included in the analysis. Discriminant validity was assessed using the heterotrait-monotrait ratio (HTMT_85_ (HTMT<0.85)) as the more conservative approach and HTMT_90_ (HTMT<90) and HTMT_inference_ (HTMT <1) as the less conservative approach).

Step 2: A bootstrapping procedure with the bias-corrected and accelerated (BCa) bootstrap and 5,000 iterations were conducted to estimate confidence intervals, standard errors and to compute inference statistics (t-values=t-statistics).

**The inner model (structural)**

Step 3: The assessment of the structural model was performed looking at the collinearity among constructs, using the variance inflation factor (VIF) which is indicating the level of collinearity; for example, when two constructs are highly correlated. The critical value of VIF is 5, but when it is >3, it requires cautions as well. Therefore, low VIF values (<3) represent good values.

Step 4: The statistical significance of the path coefficients (standardised beta), the direction of the effect showed by the arrow, and the relevance of the path coefficients were assessed using the direct, indirect and total effect.

Step 5: The in-sample prediction was used for the entire data to estimate the model and to predict observation from the data sets, the values for this estimate were the coefficients of determination (R^2^) and their effect size (f^2^).

Step 6: The out-of-sample prediction was conducted using PLSpredict using the model estimates to predict new observation (e.g. future observation, holdout sample). PLS predict rests on the principle of K-fold cross-validation. The procedure splits the data set into K equal parts (K=10 in our case) and estimate the model K-times on K-1 data sets using r as the number of repetition (r=10 in our case because it is a good trade-off between accuracy and running time). In this case if the predictive relevance (Q^2^) >0 the model outperforms the most naive benchmark such as the comparison between the root mean squared error of prediction (RMSE) values obtained with PLSpredict versus the RMSE values obtained with a linear model (LM). If the RMSE (PLS) yields higher prediction errors in terms of RMSE for all values this means no predictive power, the majority (low predictive power), the minority or the same (medium predictive power) none of the indicators (high predictive power).
